# Supplementary material for: The Adenylate Cyclase (CyaA) Toxin from Bordetella pertussis Has No Detectable Phospholipase A (PLA) Activity In Vitro
Source: Toxins (Basel). 2019 Feb 13;11(2):111. doi: 10.3390/toxins11020111 (PMC6409671; doi:10.3390/toxins11020111)
Supplement: Supplementary file 1 [file toxins-11-00111-s001.zip › toxins-445408 supplementary final/toxins-445408 supplementary.docx]

Supplementary Materials: The Adenylate Cyclase (CyaA) Toxin from *Bordetella pertussis* Has No Detectable Phospholipase A (PLA) Activity in Vitro

Alexis Voegele, Mirko Sadi, Dorothée Raoux-Barbot, Thibaut Douché, Mariette Matondo, Daniel Ladant and Alexandre Chenal

**Figure S1.** Phospholipase assay using PED6 as fluorogenic substrate for PLA2 activity. LUV made of DOPC: PED6 at a molar ratio 8:2 were incubated at 37 °C under constant stirring in (**A**) buffer 20 mM HEPES, 150 mM NaCl, 10 mM CaCl_2_ pH 7,4, completed with: (**B**) urea at 60 mM final concentration, (**C**) BSA at 10 nM, (**D**) Crotoxin at 10 nM, (**E**) CyaA_IP_ at 10 nM and (**F**) CyaA_UBC_ at 10 nM. Excitation was set at 480 nm and fluorescence emission spectra were recorded from 490 to 650 nm, every minute for 30 min.

**Figure S2.** Phospholipase assay using B3781 as fluorogenic substrate for PLA2 activity. LUV made of POPC: POPG: B3781 79:20:1 were incubated at 37 °C under constant stirring in presence of (**A**) Crotoxin at 100 nM, (**B**) CyaA_IP_ at 600 nM, (**C**) urea at 180 mM final concentration. Excitation was set at 342 nm and fluorescence emission spectra were recorded from 350 to 600 nm, every minute for 30 min. The excimer band at 480 nm decreases while the monomeric bands (in between 360–400 nm) of acylated pyrene derivatives increases over the time course of the experiment.
